# Spatiotemporal Dynamics of the HIV-1 Subtype G Epidemic in West and Central Africa

**DOI:** 10.1371/journal.pone.0098908

**Published:** 2014-06-11

**Authors:** Edson Delatorre, Daiana Mir, Gonzalo Bello

**Affiliations:** Laboratório de AIDS & Imunologia Molecular, Instituto Oswaldo Cruz, FIOCRUZ, Rio de Janeiro, Brazil; University of Athens, Medical School, Greece

## Abstract

The human immunodeficiency virus type 1 (HIV-1) subtype G is the second most prevalent HIV-1 clade in West Africa, accounting for nearly 30% of infections in the region. There is no information about the spatiotemporal dynamics of dissemination of this HIV-1 clade in Africa. To this end, we analyzed a total of 305 HIV-1 subtype G *pol* sequences isolated from 11 different countries from West and Central Africa over a period of 20 years (1992 to 2011). Evolutionary, phylogeographic and demographic parameters were jointly estimated from sequence data using a Bayesian coalescent-based method. Our analyses indicate that subtype G most probably emerged in Central Africa in 1968 (1956–1976). From Central Africa, the virus was disseminated to West and West Central Africa at multiple times from the middle 1970s onwards. Two subtype G strains probably introduced into Nigeria and Togo between the middle and the late 1970s were disseminated locally and to neighboring countries, leading to the origin of two major western African clades (G_WA-I_ and G_WA-II_). Subtype G clades circulating in western and central African regions displayed an initial phase of exponential growth followed by a decline in growth rate since the early/middle 1990s; but the mean epidemic growth rate of G_WA-I_ (0.75 year^−1^) and G_WA-II_ (0.95 year^−1^) clades was about two times higher than that estimated for central African lineages (0.47 year^−1^). Notably, the overall evolutionary and demographic history of G_WA-I_ and G_WA-II_ clades was very similar to that estimated for the CRF06_cpx clade circulating in the same region. These results support the notion that the spatiotemporal dissemination dynamics of major HIV-1 clades circulating in western Africa have probably been shaped by the same ecological factors.

## Introduction

The current distribution of human immunodeficiency virus type 1 (HIV-1) group M subtypes and circulating recombinant forms (CRFs) around the world resulted from the chance exportation of different viral strains out of Central Africa into new geographic regions were these initiated secondary epidemics [1]. A recent study suggests that spatial accessibility (human migrations and movements through transportation link availability and quality) has played a significant role in HIV-1 spread across sub-Saharan Africa and may explain the heterogeneous distribution of HIV-1 subtypes and CRFs in the different African regions [2].

West Africa is one of the most strongly connected regions in the continent [2] and also appears as an area of intense intra-regional migration [3]. This coincides with an overall dominance of the CRF02_AG variant, that accounts for about 50% of all HIV-1 infections in West Africa [4]. A closer inspection of the HIV-1 molecular epidemiological profile in this African region, however, reveals an important intra-regional heterogeneity in the distribution of other viral clades, including subtype G and CRF06_cpx. Subtype G is the second most prevalent HIV-1 clade in West Africa accounting for nearly 30% of infections in the region [4]. Its prevalence greatly varies within and between countries, comprising 30–50% of HIV-1 infections across different regions from Nigeria [5], [6], [7], [8], [9], [10], [11], [12], 5–15% in Benin, Niger and Togo [13], [14], [15], [16], [17], and ≤4% in other western African countries [14], [18], [19], [20], [21], [22], [23], [24], [25], [26], [27], [28], [29], [30]. Similarly, the occurrence of the CRF06_cpx clade ranges from 40–50% of HIV-1 infections in Burkina Faso [18], [19], [20], to 5–15% in Benin, Ghana, Mali, Niger, Nigeria, Senegal and Togo [5], [6], [7], [8], [9], [10], [11], [12], [13], [14], [15], [16], [17], [21], [22], [23], [24], [28], [29], and <3% in other western African countries [14], [26], [27], [30].

The highly heterogeneous distribution of subtype G and CRF06_cpx across the well-connected western African countries, suggests that spatial accessibility is not enough to fully explain the spatial distribution of those HIV-1 clades in this African region. A recent study conducted by our group suggests that Burkina Faso was the most important epicenter of dissemination of the HIV-1 CRF06_cpx strain at regional level and that CRF06_cpx prevalence decreases exponentially as we move away from the epicenter [31]. Our study also estimated that the CRF06_cpx clade started to spread in West Africa around the late 1970s [31], almost 10 years later than the estimated origin of the CRF02_AG clade in West Central Africa [32]. We postulated that the relatively late introduction of the CRF06_cpx clade into western Africa combined with the stabilization of the HIV epidemic in several countries from the region since the early/middle 1990s may have resulted in a more limited dissemination away from the epicenter and a more heterogeneous regional distribution of CRF06_cpx when compared with CRF02_AG.

It is unclear whether this hypothesis could also explain the complex distribution of subtype G in West Africa. The objective of this study was to reconstruct the onset date, dissemination routes and demographic history of the HIV-1 subtype G clade in the African continent. To this end, we used a Bayesian coalescent-based framework to analyze 305 HIV-1 subtype G *pol* sequences isolated from 11 different countries from West (Benin, Ghana, Nigeria, Senegal and Togo), West Central (Cameroon, Equatorial Guinea and Gabon), and Central Africa (Angola, Democratic Republic of Congo and Republic of Congo) over a period of 20 years (1992 to 2011).

## Materials and Methods

### Sequence Dataset

All HIV-1 subtype G *pol* sequences from West and Central African countries that covered the entire protease and partial reverse transcriptase (PR/RT) regions (nt 2253–3272 relative to HXB2 clone) and for which the sampling year was known, were downloaded from the Los Alamos HIV Sequence Database (www.hiv.lanl.gov) by August 2013. The subtype assignment of all sequences was confirmed by: REGA HIV subtyping tool v.2 [33], Maximum Likelihood (ML) phylogenetic analysis, and bootscanning analysis. A ML phylogeny with HIV-1 group M subtype reference sequences was constructed with the PhyML 3.0 program [34] using an online web server [35]. The ML tree was inferred under the GTR+I+G nucleotide substitution model recommended by the jModeltest program [36]. The heuristic tree search was performed using the SPR branch-swapping algorithm and branch support was calculated with the approximate likelihood-ratio (aLRT) SH-like test [37]. In bootscanning analyses, supporting branching of query sequences with HIV-1 group M subtypes reference sequences was determined in Neighbor-Joining trees constructed with the Kimura two-parameter model, within a 250 bp window moving in steps of 10 bases, using Simplot software v.3.5.1 [38]. We detected that 4.7% of the subtype G *pol* sequences available in database had incorrect subtype classification, consistent with previous estimations [39]. Sequences with incorrect classification, multiple sequences from the same individual and sequences from countries poorly represented (*n*<4 sequences) were removed, resulting in a final data set of 305 HIV-1 subtype G *pol* African sequences (Table 1). All codon positions known to be associated with major antiretroviral drug resistance were maintained in the final alignment because ML trees constructed on alignments with or without such positions resulted in the same overall topology (data not shown). Final sequence alignment is available from the authors upon request.

**Table 1:** HIV-1 subtype G *pol* dataset.

Region	Country	Location^a^	*N*	Sampling interval
West Africa	Benin	BJ	15	2004–2009
	Ghana	TG/GH	8	2002–2009
	Nigeria	NG	183	1992–2010
	Senegal	SN	12	1998–2010
	Togo	TG/GH	13	2006–2008
West Central Africa	Cameroon	CM	31	1997–2011
	Gabon	GA/GQ	6	2000–2008
	Equatorial Guinea	GA/GQ	4	2005–2009
Central Africa	Angola	AO/CD/CG	13	1997–2010
	DRC	AO/CD/CG	12	1993–2007
	Republic of Congo	AO/CD/CG	8	2003

a Location assigned in the Bayesian phylogeographic analysis. DRC: Democratic Republic of Congo.

### Analysis of Spatiotemporal Dispersion Pattern and Demographic History

The evolutionary rate (*µ*, nucleotide substitutions per site per year, subst./site/year), the age of the most recent common ancestor (T_MRCA_, years), the ancestral geographic movements, and the mode and rate (*r*, years-1) of population growth of HIV-1 subtype G clades circulating in Africa were jointly estimated using the Bayesian Markov Chain Monte Carlo (MCMC) approach as implemented in BEAST v1.8 [40], [41] with BEAGLE to improve run-time [42]. Analyses were performed under a GTR+I+G nucleotide substitution model. The temporal scale of evolutionary process was estimated from the sampling dates of the sequences using a relaxed uncorrelated lognormal molecular clock model and a uniform prior on clock rate (1.0–4.0×10^−3^ subst/site/year) [43]. Migration events throughout the phylogenetic history were inferred using a reversible discrete Bayesian phylogeographic model [44], in which all possible reversible exchange rates between locations were equally likely, and a CTMC rate reference prior [45]. To quantify the dissemination process, we estimated the number of viral migrations among locations using ‘Markov Jump’ counts [46] of location-state transitions along the posterior tree distribution as previously described [47], [48]. Changes in effective population size through time were initially estimated using a flexible Bayesian Skyline coalescent model [49] that does not require strong prior assumptions of demographic history. Estimates of the population growth rate were subsequently obtained using the parametric model (logistic, exponential or expansion) that provided the best fit to the demographic signal contained in datasets. Comparison between demographic models was performed using the log marginal likelihood (ML) estimation based on path sampling (PS) and stepping-stone sampling (SS) methods [50]. MCMC chains were run for 50–500×10^6^ generations. Adequate chain mixing and uncertainty in parameter estimates were assessed by calculating the Effective Sample Size (ESS) and the 95% Highest Probability Density (HPD) values, respectively, using the TRACER v1.6 program [51]. Maximum clade credibility (MCC) trees were summarized from the posterior distribution of trees with TreeAnnotator and visualized with FigTree v1.4.0 [52]. Migratory events across time were summarized using the cross-platform SPREAD application [53].

## Results

### Origin of the HIV-1 Subtype G and Identification of Major African Clades

**Figure 1:**
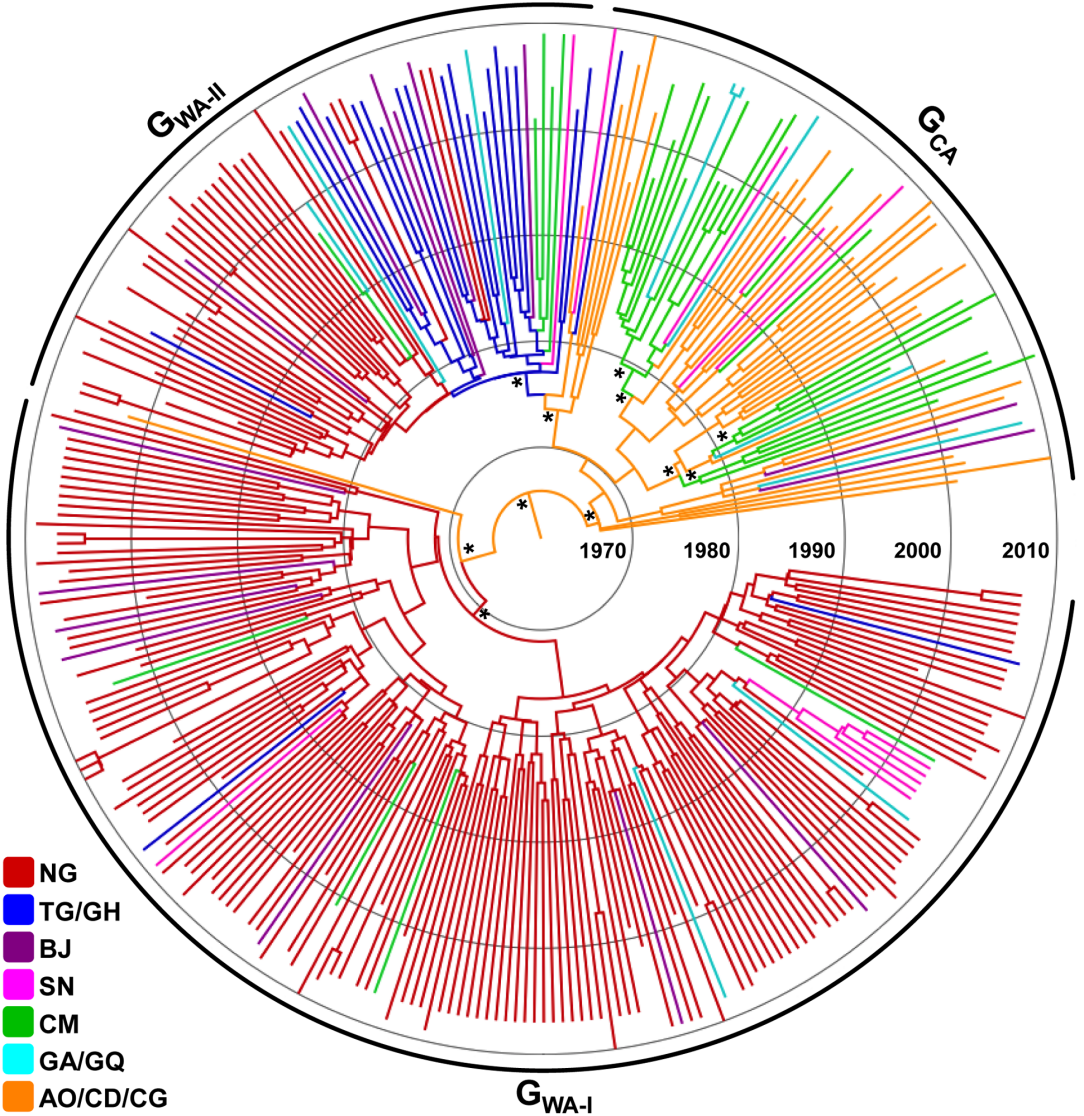
Time-scaled Bayesian MCC tree of the HIV-1 subtype G *pol* PR/RT sequences (∼1,000 nt) circulating in West and Central Africa. Branches are colored according to the most probable location state of their descendent nodes as indicated at the legend (bottom left). Arcs indicate the positions of major subtype G clades characteristic of western (G_WA-I_ and G_WA-II_) and central (G_CA_) African regions. Asterisks point to key nodes with high posterior state probability support (*PSP*>0.85). Branch lengths are drawn to scale of years. The tree was automatically rooted under the assumption of a relaxed molecular clock.

We analyzed 305 HIV-1 subtype G *pol* sequences isolated from 11 African countries between 1992 and 2011 that were sampled across seven different location states (Table 1). Neighboring countries from West (Togo/Ghana), West Central (Gabon/Equatorial Guinea) and Central (Angola/Democratic Republic of Congo/Republic of Congo) Africa comprising few samples (*n*<15) were grouped into the same location (Table 1). According to the Bayesian MCMC analysis, the median evolutionary rate of the HIV-1 subtype G lineage at *pol* gene was estimated at 2.3×10^−3^ (95% HPD: 1.8×10^−3^−2.8×10^−3^) subst./site/year. The estimated coefficient of rate variation in our dataset was 0.28 (95% HPD: 0.24–0.32), thus supporting a significant variation of substitution rate among branches and the use of a relaxed molecular clock model. The most probable root location of the subtype G clade was placed in Central Africa (posterior state probability, *PSP* = 0.88), and the onset date of this clade was estimated to be 1968 (95% HPD: 1956–1976) (Fig. 1).

**Figure 2:**
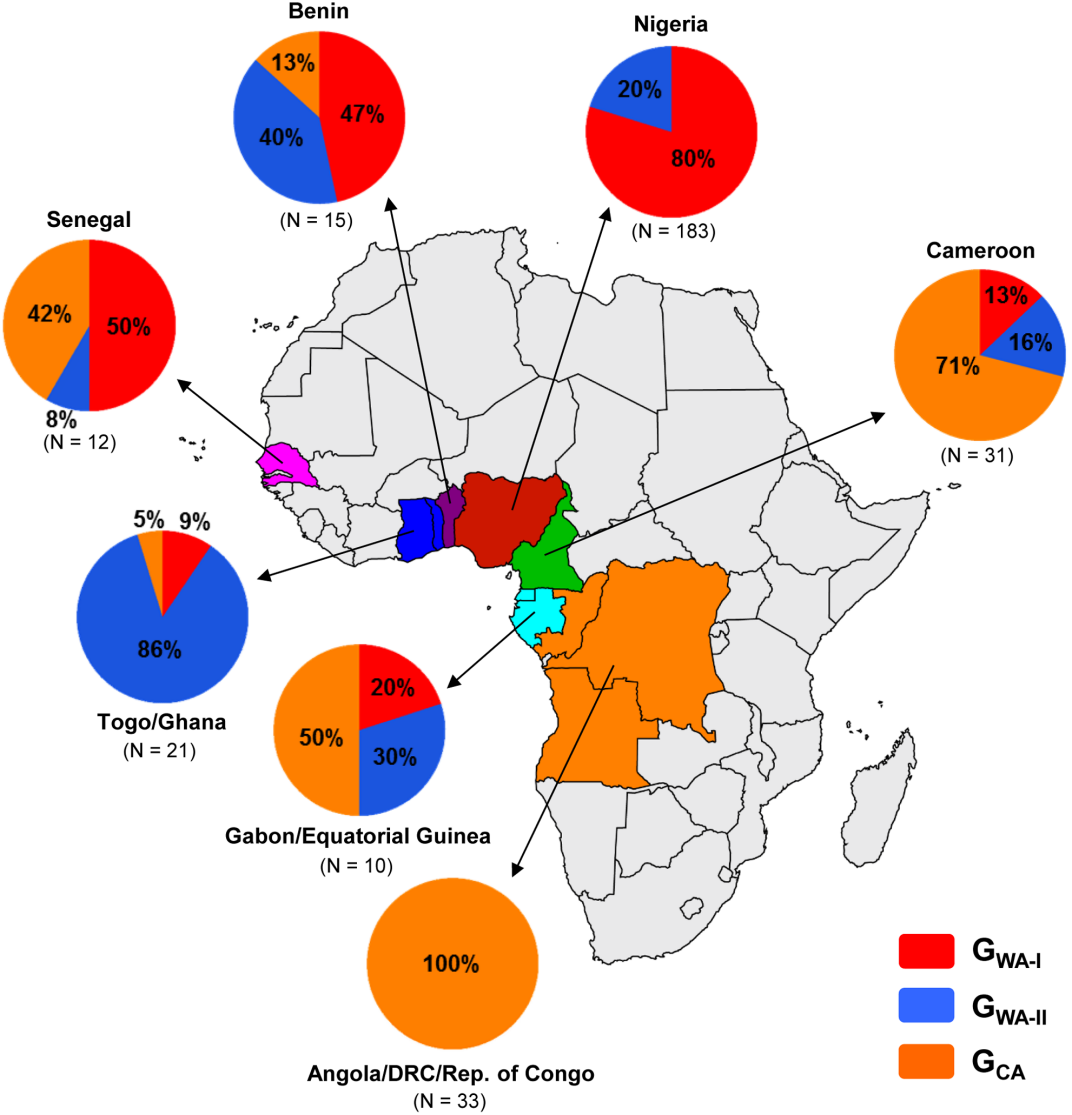
Prevalence of G_WA-I_, G_WA-II_ and G_CA_ clades among subtype G infected individuals from different African countries, estimated from phylogenetic analyses presented in Figs. 1 and S1. The total number of subtype G sequences analyzed in each locality is indicated. Each clade is represented by a color as indicated at the legend.

The Bayesian MCC (Fig. 1) and ML (Fig. S1) trees point to a clear phylogeographic subdivision of subtype G strains from West and Central Africa. Sequences from western Africa branched mostly in two large monophyletic clades (G_WA-I_ and G_WA-II_) that were nested among the most basal clades from Central and West Central Africa (G_CA_). Distribution of HIV-1 subtype G clades greatly varies across countries within each region (Fig. 2). The G_WA-I_ clade was the predominant subtype G lineage detected in Nigeria (80%) and the G_WA-II_ clade predominates in Togo/Ghana (86%). The subtype G epidemic in Benin is dominated by both G_WA-I_ (47%) and G_WA-II_ (40%) clades, whereas G_WA-I_ (50%) and G_CA_ (42%) clades prevail among subtype G infections in Senegal. Basal G_CA_ clades predominate in countries from both central (100%) and west central (50–71%) regions.

### Spatiotemporal Dispersal Pattern of the HIV-1 Subtype G African’s Epidemic

**Figure 3:**
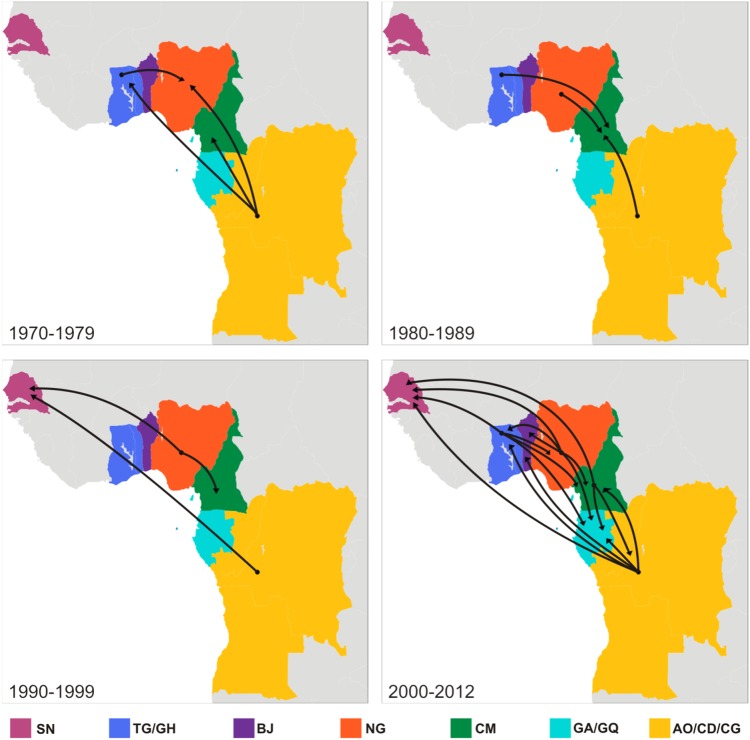
Spatiotemporal dynamics of HIV-1 subtype G clade dissemination in West and Central Africa. Snapshots of viral migration events occurring at different time intervals between 1970 and 2012 are shown. Lines between locations represent branches in the Bayesian MCC tree along which location transitions occur. Each location is represented by a color as indicated at the legend. SN: Senegal; TG/GH: Togo/Ghana; BJ: Benin; NG: Nigeria; CM: Cameroon; GA/GQ: Gabon/Equatorial Guinea; AO/CD/CG: Angola/DRC/Republic of Congo.

Reconstruction of viral migrations across time revealed the occurrence of multiple introductions of HIV-1 subtype G strains from Central into West Africa since the middle 1970s (Fig. 3). The earliest viral migrations led to the origin of the G_WA-I_ and G_WA-II_ lineages. The G_WA-I_ clade most probably emerged in Nigeria (*PSP* = 1) around 1974 (95% HPD: 1966–1981) and from this country was later disseminated to Benin, Cameroon, Equatorial Guinea, Ghana, and Senegal. The G_WA-II_ clade most probably emerged in Togo/Ghana (*PSP* = 0.68) around 1979 (95% HPD: 1973–1984) and was disseminated to Nigeria in 1981 (95% HPD: 1976–1986), where it further spread locally. In the following years, the G_WA-II_ clade was disseminated from both Togo/Ghana and Nigeria to Benin, Cameroon, Gabon, and Senegal. Our phylogeographic analysis also detected several independent introductions of subtype G variants from Central Africa into Cameroon (Figs 1 and 3). The earliest introductions occurred between the late 1970s and the middle 1980s and gave rise to at least three local Cameroonian clades; one of which was further disseminated to Gabon, Equatorial Guinea, Senegal and Angola.

**Figure 4:**
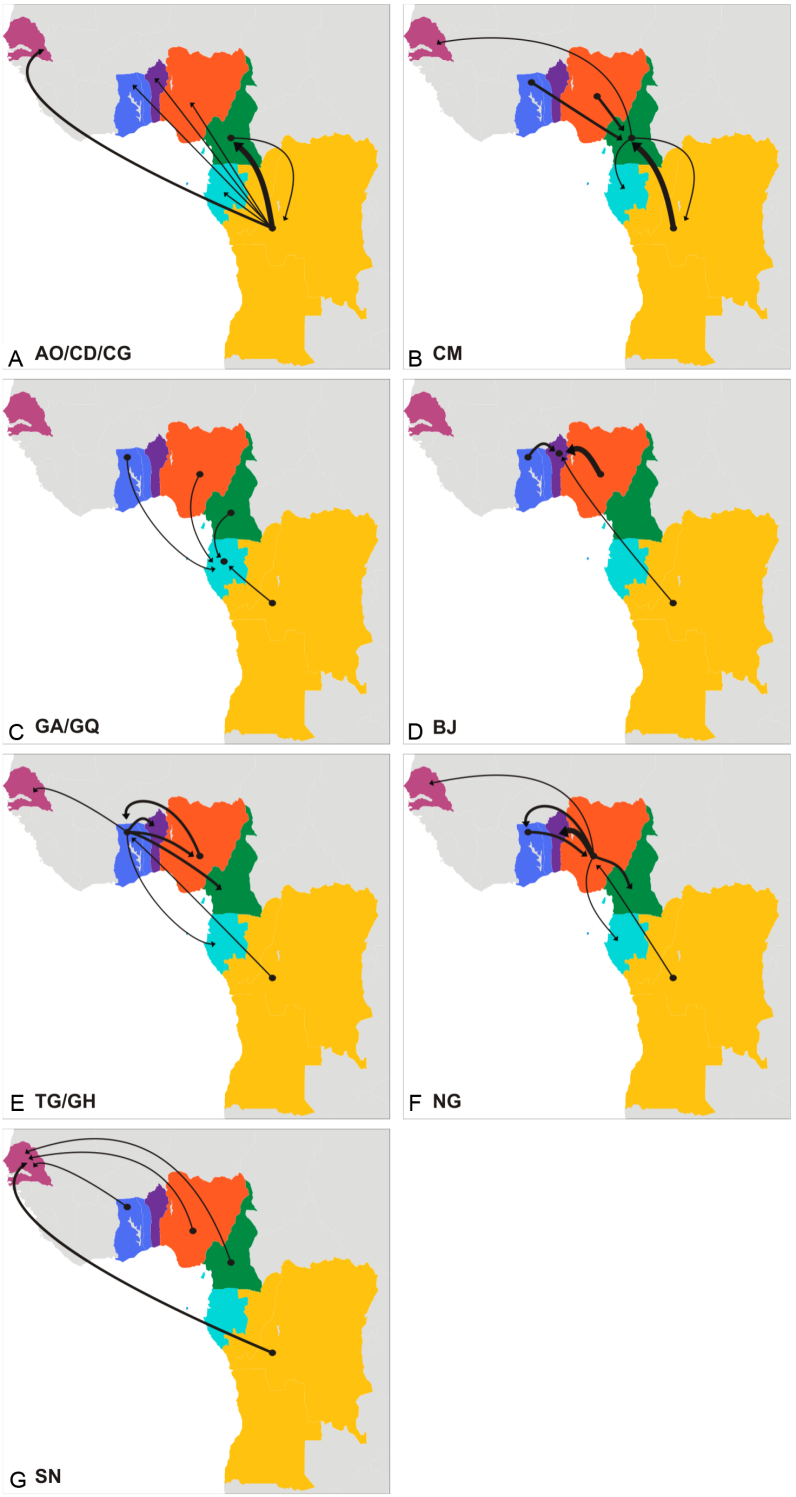
Viral migration rates among locations as measured using ‘Markov jump’ counts. Each panel represents the estimated viral exchanges from and to Angola/DRC/Republic of Congo (A), Cameroon (B), Gabon/Equatorial Guinea (C), Benin (D), Togo/Ghana (E), Nigeria (F), and Senegal (G). The width of the arrows is proportional to the corresponding mean estimated number of viral transitions between locations according to the following scale: thin arrows = 1.0–2.9 transitions, medium arrows = 3.0–5.9 transitions, thick arrows = 6.0–8.9 transitions. No arrows were displayed when the mean estimated number of transitions was below one.

We next quantified the viral flux between locations using Markov jump counts (Fig. 4 and Table S1). Nigeria (16.4), central African countries (14.8), and Togo/Ghana (8.3) displayed positive net viral migration rates (efflux minus influx), whereas Benin (−14.2), Cameroon (−10.1), Gabon/Equatorial Guinea (−8.4), and Senegal (−6.8) displayed negative net viral migration fluxes. The highest numbers of viral transitions were from Nigeria to Benin (8.1), Togo/Ghana (5.5) and Cameroon (4.4), from Central Africa to Cameroon (6.1) and Senegal (4.2), and from Togo/Ghana to Benin (5.7), Nigeria (4.1) and Cameroon (3.9). The estimated viral flux to Gabon/Equatorial Guinea from Cameroon (2.6), Central Africa (2.3), Nigeria (2.3) and Togo/Ghana (2.3) was very similar.

### Demographic History of HIV-1 Subtype G African’s Epidemic

**Figure 5:**
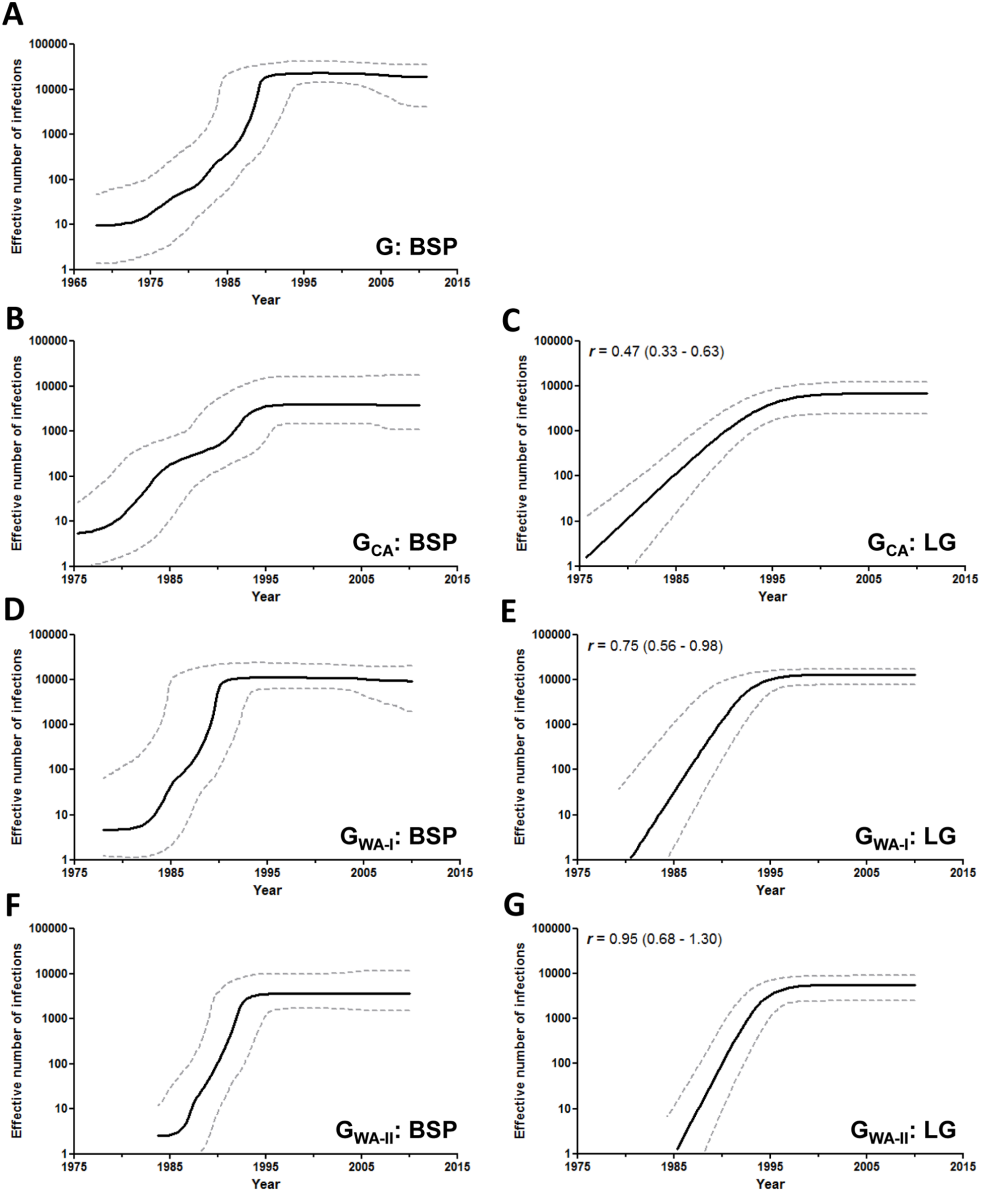
Demographic history of the HIV-1 subtype G and the clades G_CA_, G_WA-I_ and G_WA-II_ circulating in Central and West Africa. Effective number of infections (y-axis; log10 scale) through time (x-axis; calendar years) estimated using Bayesian skyline (A, B, D, F) and logistic (C, E, G) growth coalescent model. Median estimates of the effective number of infections (solid line) and 95% HPD intervals of the estimates (dashed lines) are shown in each graphic. The median growth rate (with the corresponding 95% credibility interval in parenthesis) of each clade estimated under the logistic growth model is indicated in the upper left corner.

Estimations of effective population size (*Ne*) changes over time were initially obtained using a Bayesian skyline plot (BSP) coalescent model. The BSP analysis of the complete dataset suggests that the subtype G African epidemic experienced a fast exponential growth during the 1970s and 1980s, followed by a more recent stabilization since the early 1990s (Fig. 5A). This overall growth pattern, however, represents the combined population dynamics of the different African subtype G clades that are being disseminated within different countries and regions. In order to better understand the regional differences in the demographic histories of HIV-1 subtype G African epidemics, the G_CA_, G_WA-I_ and G_WA-II_ clades were analyzed separately (Table S2).

The BSP analyses suggest that all African subtype G clades displayed a similar population growth pattern characterized by an initial phase of exponential growth followed by a decline in growth rate since the early/middle 1990s (Figs. 5B, D and F). To estimate the mean epidemic growth rate of the major subtype G African clades, log ML for the logistic, exponential and expansion growth models were calculated using both PS and SS methods. The best-fit demographic model for all subtype G clades was the logistic one (log BF>5) (Table S3) that was then used to estimate the initial epidemic growth rate. The overall time-scale and demographic pattern obtained from both BSP (Figs. 5B, D and F) and logistic growth coalescent tree priors (Figs. 5C, E and G) were very similar and important differences in the epidemic growth rate were detected across subtype G clades from West and Central Africa. According to the logistic growth coalescent model, the mean growth rate of clades G_WA-I_ (0.75 year^−1^) and G_WA-II_ (0.95 year^−1^) was about two times higher than that estimated for the clade G_CA_ (0.47 year^−1^) (Fig. 5).

## Discussion

This study indicates that the HIV-1 subtype G likely originated in Central Africa around the late 1960s. The root position of the subtype G clade is fully consistent with the most accepted model that traces the origin of all HIV-1 group M subtypes to the DRC [54], [55], [56], [57] and is also resistant to the problem of sampling bias because sequences from Central Africa represent a minor fraction (9.2%) of the total subtype G sequences included in our study. The T_MRCA_ of subtype G clade here estimated (1968: 1956–1976) is also fully consistent to that previously estimated for this subtype (1970: 1960–1978) [58]. This onset date is comparable to that estimated for subtype F (1967: 1956–1976) [59]; but more recent than that of subtypes A1 (1954: 1940–1968), C (1955: 1934–1972), and D (1947: 1938–1955) [58].

After emerging in Central Africa around the late 1960s, the HIV-1 subtype G was disseminated to West and West Central Africa a few years later (1975–1980). Our phylogeographic analysis supports the occurrence of multiple introductions of HIV-1 subtype G strains from central into the western and west central African regions. Some of the viral strains disseminated during the 1970s fueled secondary outbreaks that led to the origin of specific subtype G clades. The major subtype G clades detected in our study were the G_WA-I_ that most probably emerged in Nigeria around the middle 1970s, and the G_WA-II_ that most probably emerged in Togo or Ghana around the late 1970s. Although we grouped sequences from Togo and Ghana into one single location, the much higher prevalence of subtype G in Togo (9%) [16], [17] compared with Ghana (<1%) [23], [24] suggests that the G_WA-II_ clade probably arose in Togo. We also detected three minor subtype G clades that resulted of independent introductions of viral strains from central Africa into Cameroon between the late 1970s and the middle 1980s.

Nigeria and Togo/Nigeria were inferred as the most important epicenters of dissemination of the G_WA-I_ and G_WA-II_ clades at regional level, respectively. The G_WA-I_ clade, which corresponds to the clade previously designated G’ [5], [8], was the predominant subtype G lineage in Nigeria (80%), Senegal (50%), and Benin (47%), and also comprises a significant fraction of subtype G infections in Gabon/Equatorial Guinea (20%), Cameroon (13%) and Togo/Ghana (9%). The G_WA-II_ clade predominates in Togo/Ghana (86%) and is responsible for a significant fraction of subtype G infections in Benin (40%), Gabon/Equatorial Guinea (30%), Nigeria (20%), Cameroon (16%) and Senegal (8%). The subtype G clades introduced into Cameroon were mainly disseminated to the neighboring countries in the central west region (Gabon and Equatorial Guinea), although a few disseminations to Senegal were also detected. These results indicate that founder subtype G strains introduced into Nigeria and Togo have been much more efficiently disseminated at regional level than those introduced into Cameroon.

Our demographic reconstructions also revealed another important difference between African subtype G clades mainly disseminated in the western region (G_WA-I_ and G_WA-II_) and those mainly disseminated in the west central and central regions (G_CA_). Although all African subtype G clades displayed a similar population growth pattern characterized by an initial phase of exponential growth followed by a decline in growth rate since the early/middle 1990s; the mean epidemic growth rate of G_WA-I_ (0.75 year^−1^) and G_WA-II_ (0.95 year^−1^) clades was about two times higher than that estimated for G_CA_ (0.47 year^−1^) clades. This suggests that subtype G clades introduced into Nigeria and Togo during the 1970s probably encountered more favorable conditions for local and regional expansion than those disseminated within central and west-central African countries around the same time. The median growth rates of the G_WA-I_ and G_WA-II_ clades were comparable to that estimated for the CRF06_cpx in western Africa (0.82 year^−1^) [31]; whereas the median growth rate of the G_CA_ clades was roughly similar to that estimated for subtype G in Cuba (0.54 year^−1^) [60] and higher than that estimated for HIV-1 group M in Democratic Republic of Congo (0.17 year^−1^) [61].

The faster epidemic growth and the broader geographic dissemination of subtype G strains introduced into West Africa compared with those circulating in the central west and central African regions could be associated to clade-specific or regional-specific differences in viral transmissibility. It has been suggested that accessibility between locations have played a major role in the spatial spread of HIV-1 in sub-Saharan Africa [2]. Notably, West Africa is one of the most strongly connected regions in the continent [2] and also displays an intra-regional migration rate (3%) above the African average (2%) [3]. Others factors including urbanization [56], [62], iatrogenic interventions [63], [64], and forced migration [62], [65] might have also played a role in the emergence and spread of HIV in Africa. Such alternative scenarios can now be tested in a Bayesian framework [66] to find the hypothesis that best explain the variability in the rate of HIV spread across African regions.

Despite the strong regional accessibility, the prevalence of subtype G and CRF06_cpx clades greatly vary across western African countries. The clades CRF06_cpx, G_WA-I_, and G_WA-II_ seem to have experienced very similar dissemination dynamics; although their origin was traced to different western African countries (Burkina Faso, Nigeria and Togo, respectively) [31]. The three HIV-1 clades probably started to spread in West Africa around the same time (1975–1980), expanded during the 1980s with similar epidemic growth rates (0.75–0.95 year^−1^), started to stabilize around the early/middle 1990s, and their prevalence is greatly reduced as we moved away from the corresponding epicenters [31]. The relatively late spread of subtype G and CRF06_cpx clades in West Africa combined with: 1) stabilization of the HIV epidemic in several western African countries since the early/middle 1990s, and/or 2) depletion of the susceptible populations most at risk by the firstly introduced CRF02_AG lineage, may have limited the dissemination of these viral clades far from the epicenter, thus generating a heterogeneous spatial distribution.

The most important limitation of our study was the small sampling size of many African countries. Only Nigeria (*n* = 183) and Cameroon (*n* = 31) were represented by a high or relatively high number of sequences. Other western (Benin, Niger, and Togo) and central (Central African Republic, Chad, Equatorial Guinea, and Gabon) African countries with circulation of subtype G at significant levels (≥5% of all HIV-1 infections) [13], [14], [15], [16], [17], [67], [68], [69], [70], [71], [72] were represented by a small number of sequences (*n*≤15) that may not fully reflect the country’s subtype G diversity, or were not represented at all in our study (Fig. S2). Thus, a more comprehensive and balanced sampling from countries poorly or not represented here would certainly provide more precise estimates of the relative prevalence and migration routes of clades G_WA-I_, G_WA-II_ and G_CA_ across different African regions, and may also result in the identification of new regional viral clades not detected in this study.

It will be also interesting to trace the origins and global dispersal pathways of those subtype G lineages found in countries outside sub-Saharan Africa, particularly in Cuba [73], [74], [75], Portugal [76], [77], [78], and Russia [79] where this subtype has been disseminated among local populations. It has been showed that the spread of HIV-2 outwards Africa mirrors socio historical ties [80] and a previous study conducted by our group showed that most subtype G Cuban lineages are nested among basal sequences from Central Africa [60]. Thus, circulation of subtype G outside sub-Saharan Africa may be linked to the presence of Portuguese, Cuban, and Russian personnel in Angola and neighboring countries during 1960–1990.

In summary, this study suggests that the HIV-1 subtype G clade started to circulate in Central Africa around the late 1960s and was disseminated to West and West Central Africa from the middle 1970s onwards. Nigeria and Togo were pointed out as the major secondary hubs of dissemination of subtype G within western and west central African regions. Our data also highlight that the spatiotemporal dissemination dynamics of western African subtype G clades were very similar to that estimated for the CRF06_cpx epidemic; supporting the notion that current distribution of major HIV-1 clades in West Africa may have been shaped by the same ecological factors. Despite some study limitations, these findings offer important insights toward an understanding of the current characteristics and dynamics of the HIV-1 epidemic in West and West Central Africa.

## Supporting Information

Figure S1 **ML tree of the of the HIV-1 subtype G** ***pol*** **PR/RT sequences (∼1,000 nt) circulating in West and Central Africa.** Branches are colored according to the geographic origin of each sequence as indicated at the legend (bottom left). Arcs indicate the positions of major subtype G clades characteristic of western (G_WA-I_ and G_WA-II_) and central (G_CA_) African regions. Asterisks point to key nodes with high support (*a*LRT>0.85). The tree was rooted on midpoint. The branch lengths are drawn to scale with the bar at the bottom indicating nucleotide substitutions per site.
(PDF)

Figure S2 **African map showing the prevalence of subtype G among HIV-1-infected individuals from West and West Central Africa, and the corresponding representativeness of each African country in our subtype G dataset.** Countries were colored according to the relative prevalence of subtype G (estimated from references 5–30 and 53–58) as shown in the legend. Asterisks indicate countries represented by very high (***n>100), relatively high (**n>30), and small (*n≤30) number of sequences. Countries with no asterisks were not represented in our dataset.
(PDF)

Table S1 **Number of viral migration between locations estimated using Markov jumps counts.**
(PDF)

Table S2 **Evolutionary rate and time-scale of HIV-1 subtype G and major regional clades circulating in Africa.**
(PDF)

Table S3 **Best fit demographic model for HIV-1 subtype G African clades.**
(PDF)

## References

[1] Archer J, Robertson DL (2007) Understanding the diversification of HIV-1 groups M and O. Aids. 21: 1693–1700. 10.1097/QAD.0b013e32825eabd0 17690566

[2] Tatem AJ, Hemelaar J, Gray RR, Salemi M (2012) Spatial accessibility and the spread of HIV-1 subtypes and recombinants in sub-Saharan Africa. AIDS 26: 2351–2360. 22951637 10.1097/QAD.0b013e328359a904

[3] Gnisci D, Trémolières M, (SWAC/OECD) (2009) West African Studies. Regional Atlas on West Africa. Population and settlement. Charpter 4. Migration. OECD Publishing: 67–85.

[4] Hemelaar J, Gouws E, Ghys PD, Osmanov S (2011) Global trends in molecular epidemiology of HIV-1 during 2000–2007. Aids 25: 679–689. 21297424 10.1097/QAD.0b013e328342ff93 PMC3755761

[5] Peeters M, Esu-Williams E, Vergne L, Montavon C, Mulanga-Kabeya C, et al (2000) Predominance of subtype A and G HIV type 1 in Nigeria, with geographical differences in their distribution. AIDS Res Hum Retroviruses 16: 315–325. 10716369 10.1089/088922200309197

[6] Agwale SM, Zeh C, Robbins KE, Odama L, Saekhou A, et al (2002) Molecular surveillance of HIV-1 field strains in Nigeria in preparation for vaccine trials. Vaccine 20: 2131–2139. 11972982 10.1016/s0264-410x(02)00059-2

[7] Ojesina AI, Sankale JL, Odaibo G, Langevin S, Meloni ST, et al (2006) Subtype-specific patterns in HIV Type 1 reverse transcriptase and protease in Oyo State, Nigeria: implications for drug resistance and host response. AIDS Res Hum Retroviruses 22: 770–779. 16910833 10.1089/aid.2006.22.770

[8] Sankale JL, Langevin S, Odaibo G, Meloni ST, Ojesina AI, et al (2007) The complexity of circulating HIV type 1 strains in Oyo state, Nigeria. AIDS Res Hum Retroviruses 23: 1020–1025. 17725419 10.1089/aid.2006.0304

[9] Chaplin B, Eisen G, Idoko J, Onwujekwe D, Idigbe E, et al (2011) Impact of HIV type 1 subtype on drug resistance mutations in Nigerian patients failing first-line therapy. AIDS Res Hum Retroviruses 27: 71–80. 20964479 10.1089/aid.2010.0050 PMC3034099

[10] Hamers RL, Wallis CL, Kityo C, Siwale M, Mandaliya K, et al (2011) HIV-1 drug resistance in antiretroviral-naive individuals in sub-Saharan Africa after rollout of antiretroviral therapy: a multicentre observational study. Lancet Infect Dis 11: 750–759. 21802367 10.1016/S1473-3099(11)70149-9

[11] Ajoge HO, Gordon ML, Ibrahim S, Shittu OS, Ndung’u T, et al (2012) Drug resistance pattern of HIV type 1 isolates sampled in 2007 from therapy-naive pregnant women in North-Central Nigeria. AIDS Res Hum Retroviruses 28: 115–118. 21568761 10.1089/aid.2011.0115

[12] Imade GE, Sagay AS, Chaplin B, Chebu P, Musa J, et al (2014) Short communication: Transmitted HIV drug resistance in antiretroviral-naive pregnant women in north central Nigeria. AIDS Res Hum Retroviruses 30: 127–133. 24164431 10.1089/aid.2013.0074 PMC3910445

[13] Mamadou S, Montavon C, Ben A, Djibo A, Rabiou S, et al (2002) Predominance of CRF02-AG and CRF06-cpx in Niger, West Africa. AIDS Res Hum Retroviruses 18: 723–726. 12167280 10.1089/088922202760072357

[14] Charpentier C, Bellecave P, Cisse M, Mamadou S, Diakite M, et al (2011) High prevalence of antiretroviral drug resistance among HIV-1-untreated patients in Guinea-Conakry and in Niger. Antivir Ther 16: 429–433. 21555827 10.3851/IMP1754

[15] Chamberland A, Diabate S, Sylla M, Anagounou S, Geraldo N, et al (2012) Transmission of HIV-1 drug resistance in Benin could jeopardise future treatment options. Sex Transm Infect 88: 179–183. 22158948 10.1136/sextrans-2011-050209

[16] Yaotse DA, Nicole V, Roch NF, Mireille PD, Eric D, et al (2009) Genetic characterization of HIV-1 strains in Togo reveals a high genetic complexity and genotypic drug-resistance mutations in ARV naive patients. Infect Genet Evol 9: 646–652. 19460333 10.1016/j.meegid.2009.04.002

[17] Dagnra AY, Vidal N, Mensah A, Patassi A, Aho K, et al (2011) High prevalence of HIV-1 drug resistance among patients on first-line antiretroviral treatment in Lome, Togo. J Int AIDS Soc 14: 30. 21663632 10.1186/1758-2652-14-30 PMC3125306

[18] Ouedraogo-Traore R, Montavon C, Sanou T, Vidal N, Sangare L, et al (2003) CRF06-cpx is the predominant HIV-1 variant in AIDS patients from Ouagadougou, the capital city of Burkina Faso. AIDS 17: 441–442. 12556699 10.1097/00002030-200302140-00019

[19] Vergne L, Diagbouga S, Kouanfack C, Aghokeng A, Butel C, et al (2006) HIV-1 drug-resistance mutations among newly diagnosed patients before scaling-up programmes in Burkina Faso and Cameroon. Antivir Ther 11: 575–579. 16964825

[20] Tebit DM, Sangare L, Tiba F, Saydou Y, Makamtse A, et al (2009) Analysis of the diversity of the HIV-1 pol gene and drug resistance associated changes among drug-naive patients in Burkina Faso. J Med Virol 81: 1691–1701. 19697403 10.1002/jmv.21600

[21] Derache A, Maiga AI, Traore O, Akonde A, Cisse M, et al (2008) Evolution of genetic diversity and drug resistance mutations in HIV-1 among untreated patients from Mali between 2005 and 2006. J Antimicrob Chemother 62: 456–463. 18556706 10.1093/jac/dkn234

[22] Maiga AI, Fofana DB, Maiga AC, Diallo F, Arkoub ZA, et al (2012) Transmitted Antiretroviral Drug Resistance in Newly HIV-Infected and Untreated Patients in Segou and Bamako, Mali. AIDS Res Hum Retroviruses. 10.1089/aid.2012.0118 PMC3537298 22823755

[23] Fischetti L, Opare-Sem O, Candotti D, Sarkodie F, Lee H, et al (2004) Molecular epidemiology of HIV in Ghana: dominance of CRF02_AG. J Med Virol 73: 158–166. 15122787 10.1002/jmv.20070

[24] Delgado E, Ampofo WK, Sierra M, Torpey K, Perez-Alvarez L, et al (2008) High prevalence of unique recombinant forms of HIV-1 in Ghana: molecular epidemiology from an antiretroviral resistance study. J Acquir Immune Defic Syndr 48: 599–606. 18645511 10.1097/QAI.0b013e3181806c0e

[25] Nii-Trebi NI, Ibe S, Barnor JS, Ishikawa K, Brandful JA, et al (2013) HIV-1 drug-resistance surveillance among treatment-experienced and -naive patients after the implementation of antiretroviral therapy in Ghana. PLoS One 8: e71972. 23977189 10.1371/journal.pone.0071972 PMC3747072

[26] Ayouba A, Lien TT, Nouhin J, Vergne L, Aghokeng AF, et al (2009) Low prevalence of HIV type 1 drug resistance mutations in untreated, recently infected patients from Burkina Faso, Cote d’Ivoire, Senegal, Thailand, and Vietnam: the ANRS 12134 study. AIDS Res Hum Retroviruses 25: 1193–1196. 19886834 10.1089/aid.2009.0142

[27] Toni T, Masquelier B, Minga A, Anglaret X, Danel C, et al (2007) HIV-1 antiretroviral drug resistance in recently infected patients in Abidjan, Cote d’Ivoire: A 4-year survey, 2002–2006. AIDS Res Hum Retroviruses 23: 1155–1160. 17919113 10.1089/aid.2007.0072

[28] Hamel DJ, Sankale JL, Eisen G, Meloni ST, Mullins C, et al (2007) Twenty years of prospective molecular epidemiology in Senegal: changes in HIV diversity. AIDS Res Hum Retroviruses 23: 1189–1196. 17961103 10.1089/aid.2007.0037

[29] Diop-Ndiaye H, Toure-Kane C, Leye N, Ngom-Gueye NF, Montavon C, et al (2010) Antiretroviral drug resistance mutations in antiretroviral-naive patients from Senegal. AIDS Res Hum Retroviruses 26: 1133–1138. 20849302 10.1089/aid.2009.0295

[30] Esbjornsson J, Mild M, Mansson F, Norrgren H, Medstrand P (2011) HIV-1 molecular epidemiology in Guinea-Bissau, West Africa: origin, demography and migrations. PLoS One 6: e17025. 21365013 10.1371/journal.pone.0017025 PMC3041826

[31] Delatorre E, Bello G (2013) Spatiotemporal dynamics of the HIV-1 CRF06_cpx epidemic in western Africa. AIDS 27: 1313–1320. 23343915 10.1097/QAD.0b013e32835f1df4

[32] Faria NR, Suchard MA, Abecasis A, Sousa JD, Ndembi N, et al (2012) Phylodynamics of the HIV-1 CRF02_AG clade in Cameroon. Infect Genet Evol 12: 453–460. 21565285 10.1016/j.meegid.2011.04.028 PMC4677783

[33] de Oliveira T, Deforche K, Cassol S, Salminen M, Paraskevis D, et al (2005) An automated genotyping system for analysis of HIV-1 and other microbial sequences. Bioinformatics 21: 3797–3800. 16076886 10.1093/bioinformatics/bti607

[34] Guindon S, Dufayard JF, Lefort V, Anisimova M, Hordijk W, et al (2010) New algorithms and methods to estimate maximum-likelihood phylogenies: assessing the performance of PhyML 3.0. Syst Biol 59: 307–321. 20525638 10.1093/sysbio/syq010

[35] Guindon S, Lethiec F, Duroux P, Gascuel O (2005) PHYML Online–a web server for fast maximum likelihood-based phylogenetic inference. Nucleic Acids Res 33: W557–559. 15980534 10.1093/nar/gki352 PMC1160113

[36] Posada D (2008) jModelTest: phylogenetic model averaging. Mol Biol Evol 25: 1253–1256. 18397919 10.1093/molbev/msn083

[37] Anisimova M, Gascuel O (2006) Approximate likelihood-ratio test for branches: A fast, accurate, and powerful alternative. Syst Biol 55: 539–552. 16785212 10.1080/10635150600755453

[38] Lole KS, Bollinger RC, Paranjape RS, Gadkari D, Kulkarni SS, et al (1999) Full-length human immunodeficiency virus type 1 genomes from subtype C-infected seroconverters in India, with evidence of intersubtype recombination. J Virol 73: 152–160. 9847317 10.1128/jvi.73.1.152-160.1999 PMC103818

[39] Zhang M, Foley B, Schultz AK, Macke JP, Bulla I, et al (2010) The role of recombination in the emergence of a complex and dynamic HIV epidemic. Retrovirology 7: 25. 20331894 10.1186/1742-4690-7-25 PMC2855530

[40] Drummond AJ, Nicholls GK, Rodrigo AG, Solomon W (2002) Estimating mutation parameters, population history and genealogy simultaneously from temporally spaced sequence data. Genetics 161: 1307–1320. 12136032 10.1093/genetics/161.3.1307 PMC1462188

[41] Drummond AJ, Rambaut A (2007) BEAST: Bayesian evolutionary analysis by sampling trees. BMC Evol Biol 7: 214. 17996036 10.1186/1471-2148-7-214 PMC2247476

[42] Suchard MA, Rambaut A (2009) Many-core algorithms for statistical phylogenetics. Bioinformatics 25: 1370–1376. 19369496 10.1093/bioinformatics/btp244 PMC2682525

[43] Drummond AJ, Ho SY, Phillips MJ, Rambaut A (2006) Relaxed phylogenetics and dating with confidence. PLoS Biol 4: e88. 16683862 10.1371/journal.pbio.0040088 PMC1395354

[44] Lemey P, Rambaut A, Drummond AJ, Suchard MA (2009) Bayesian phylogeography finds its roots. PLoS Comput Biol 5: e1000520. 19779555 10.1371/journal.pcbi.1000520 PMC2740835

[45] Ferreira MAR, Suchard MA (2008) Bayesian analysis of elapsed times in continuous-time Markov chains. Canadian Journal of Statistics 26: 355–368.

[46] Minin VN, Suchard MA (2008) Counting labeled transitions in continuous-time Markov models of evolution. J Math Biol 56: 391–412. 17874105 10.1007/s00285-007-0120-8

[47] Talbi C, Lemey P, Suchard MA, Abdelatif E, Elharrak M, et al (2010) Phylodynamics and human-mediated dispersal of a zoonotic virus. PLoS Pathog 6: e1001166. 21060816 10.1371/journal.ppat.1001166 PMC2965766

[48] Nunes MR, Faria NR, Vasconcelos HB, Medeiros DB, Silva de Lima CP, et al (2012) Phylogeography of dengue virus serotype 4, Brazil, 2010–2011. Emerg Infect Dis 18: 1858–1864. 23092706 10.3201/eid1811.120217 PMC3559147

[49] Drummond AJ, Rambaut A, Shapiro B, Pybus OG (2005) Bayesian coalescent inference of past population dynamics from molecular sequences. Mol Biol Evol 22: 1185–1192. 15703244 10.1093/molbev/msi103

[50] Suchard MA, Weiss RE, Sinsheimer JS (2001) Bayesian selection of continuous-time Markov chain evolutionary models. Mol Biol Evol 18: 1001–1013. 11371589 10.1093/oxfordjournals.molbev.a003872

[51] Rambaut A, Drummond A (2007) Tracer v1.6. Available from: http://tree.bio.ed.ac.uk/software/tracer. Accessed 2014 Apr 25.

[52] Rambaut A (2009) FigTree v1.4: Tree Figure Drawing Tool. Available from: http://tree.bio.ed.ac.uk/software/figtree/. Accessed 2014 April 25.

[53] Bielejec F, Rambaut A, Suchard MA, Lemey P (2011) SPREAD: spatial phylogenetic reconstruction of evolutionary dynamics. Bioinformatics 27: 2910–2912. 21911333 10.1093/bioinformatics/btr481 PMC3187652

[54] Rambaut A, Robertson DL, Pybus OG, Peeters M, Holmes EC (2001) Human immunodeficiency virus. Phylogeny and the origin of HIV-1. Nature 410: 1047–1048. 11323659 10.1038/35074179

[55] Vidal N, Peeters M, Mulanga-Kabeya C, Nzilambi N, Robertson D, et al (2000) Unprecedented degree of human immunodeficiency virus type 1 (HIV-1) group M genetic diversity in the Democratic Republic of Congo suggests that the HIV-1 pandemic originated in Central Africa. J Virol 74: 10498–10507. 11044094 10.1128/jvi.74.22.10498-10507.2000 PMC110924

[56] Worobey M, Gemmel M, Teuwen DE, Haselkorn T, Kunstman K, et al (2008) Direct evidence of extensive diversity of HIV-1 in Kinshasa by 1960. Nature 455: 661–664. 18833279 10.1038/nature07390 PMC3682493

[57] Kalish ML, Robbins KE, Pieniazek D, Schaefer A, Nzilambi N, et al (2004) Recombinant viruses and early global HIV-1 epidemic. Emerg Infect Dis 10: 1227–1234. 15324542 10.3201/eid1007.030904 PMC3323344

[58] Abecasis AB, Vandamme AM, Lemey P (2009) Quantifying differences in the tempo of human immunodeficiency virus type 1 subtype evolution. J Virol 83: 12917–12924. 19793809 10.1128/JVI.01022-09 PMC2786833

[59] Mehta SR, Wertheim JO, Delport W, Ene L, Tardei G, et al (2011) Using phylogeography to characterize the origins of the HIV-1 subtype F epidemic in Romania. Infect Genet Evol 11: 975–979. 21439403 10.1016/j.meegid.2011.03.009 PMC3104099

[60] Delatorre E, Bello G (2013) Phylodynamics of the HIV-1 epidemic in Cuba. PLoS ONE 8: e72448. 24039765 10.1371/journal.pone.0072448 PMC3767668

[61] Yusim K, Peeters M, Pybus OG, Bhattacharya T, Delaporte E, et al (2001) Using human immunodeficiency virus type 1 sequences to infer historical features of the acquired immune deficiency syndrome epidemic and human immunodeficiency virus evolution. Philos Trans R Soc Lond B Biol Sci 356: 855–866. 11405933 10.1098/rstb.2001.0859 PMC1088479

[62] Buve A, Bishikwabo-Nsarhaza K, Mutangadura G (2002) The spread and effect of HIV-1 infection in sub-Saharan Africa. Lancet 359: 2011–2017. 12076570 10.1016/S0140-6736(02)08823-2

[63] Lemey P, Pybus OG, Wang B, Saksena NK, Salemi M, et al (2003) Tracing the origin and history of the HIV-2 epidemic. Proc Natl Acad Sci U S A 100: 6588–6592. 12743376 10.1073/pnas.0936469100 PMC164491

[64] Gisselquist D (2004) Impact of long-term civil disorders and wars on the trajectory of HIV epidemics in sub-Saharan Africa. SAHARA J 1: 114–127. 17601017 10.1080/17290376.2004.9724834

[65] Salama P, Dondero TJ (2001) HIV surveillance in complex emergencies. Aids 15: S4–S12. 10.1097/00002030-200104003-00002 11421181

[66] Lemey P, Rambaut A, Bedford T, Faria N, Bielejec F, et al (2014) Unifying viral genetics and human transportation data to predict the global transmission dynamics of human influenza H3N2. PLoS Pathog 10: e1003932. 24586153 10.1371/journal.ppat.1003932 PMC3930559

[67] Marechal V, Jauvin V, Selekon B, Leal J, Pelembi P, et al (2006) Increasing HIV type 1 polymorphic diversity but no resistance to antiretroviral drugs in untreated patients from Central African Republic: a 2005 study. AIDS Res Hum Retroviruses 22: 1036–1044. 17067275 10.1089/aid.2006.22.1036

[68] Aghokeng AF, Vergne L, Mpoudi-Ngole E, Mbangue M, Deoudje N, et al (2009) Evaluation of transmitted HIV drug resistance among recently-infected antenatal clinic attendees in four Central African countries. Antivir Ther 14: 401–411. 19474474 10.1177/135965350901400313

[69] Koyalta D, Charpentier C, Beassamda J, Rey E, Si-Mohamed A, et al (2009) High frequency of antiretroviral drug resistance among HIV-infected adults receiving first-line highly active antiretroviral therapy in N’Djamena, Chad. Clin Infect Dis 49: 155–159. 19480574 10.1086/599611

[70] Djoko CF, Wolfe ND, Vidal N, Tamoufe U, Montavon C, et al (2010) HIV type 1 pol gene diversity and genotypic antiretroviral drug resistance mutations in Malabo, Equatorial Guinea. AIDS Res Hum Retroviruses 26: 1027–1031. 20718620 10.1089/aid.2010.0046 PMC2957628

[71] Caron M, Lekana-Douki SE, Makuwa M, Obiang-Ndong GP, Biba O, et al (2012) Prevalence, genetic diversity and antiretroviral drugs resistance-associated mutations among untreated HIV-1-infected pregnant women in Gabon, central Africa. BMC Infect Dis 12: 64. 22433277 10.1186/1471-2334-12-64 PMC3359209

[72] Pandrea I, Robertson DL, Onanga R, Gao F, Makuwa M, et al (2002) Analysis of partial pol and env sequences indicates a high prevalence of HIV type 1 recombinant strains circulating in Gabon. AIDS Res Hum Retroviruses 18: 1103–1116. 12396449 10.1089/088922202320567842

[73] Cuevas MT, Ruibal I, Villahermosa ML, Diaz H, Delgado E, et al (2002) High HIV-1 genetic diversity in Cuba. Aids 16: 1643–1653. 12172086 10.1097/00002030-200208160-00010

[74] Kouri V, Aleman Y, Perez L, Perez J, Fonseca C, et al (2012) High frequency of antiviral drug resistance and non-B subtypes in HIV-1 patients failing antiviral therapy in Cuba. J Clin Virol 55: 348–355. 22981617 10.1016/j.jcv.2012.08.019

[75] Machado LY, Blanco M, Dubed M, Diaz HM, Ruiz NM, et al (2012) HIV type 1 genetic diversity in newly diagnosed Cuban patients. AIDS Res Hum Retroviruses 28: 956–960. 22059433 10.1089/aid.2011.0295

[76] Esteves A, Parreira R, Venenno T, Franco M, Piedade J, et al (2002) Molecular epidemiology of HIV type 1 infection in Portugal: high prevalence of non-B subtypes. AIDS Res Hum Retroviruses 18: 313–325. 11897032 10.1089/088922202753519089

[77] Esteves A, Parreira R, Piedade J, Venenno T, Franco M, et al (2003) Spreading of HIV-1 subtype G and envB/gagG recombinant strains among injecting drug users in Lisbon, Portugal. AIDS Res Hum Retroviruses 19: 511–517. 12892060 10.1089/088922203766774568

[78] Palma AC, Araujo F, Duque V, Borges F, Paixao MT, et al (2007) Molecular epidemiology and prevalence of drug resistance-associated mutations in newly diagnosed HIV-1 patients in Portugal. Infect Genet Evol 7: 391–398. 17360244 10.1016/j.meegid.2007.01.009

[79] Bobkova M (2013) Current status of HIV-1 diversity and drug resistance monitoring in the former USSR. AIDS Rev 15: 204–212. 24192601

[80] Faria NR, Hodges-Mameletzis I, Silva JC, Rodes B, Erasmus S, et al (2012) Phylogeographical footprint of colonial history in the global dispersal of human immunodeficiency virus type 2 group A. J Gen Virol. 93: 889–899. 10.1099/vir.0.038638-0 PMC3542711 22190015

