# Supplementary figures and images for: Spatiotemporal Dynamics of the HIV-1 Subtype G Epidemic in West and Central Africa
Source: PLoS One. 2014 Jun 11;9(6):e98908. doi: 10.1371/journal.pone.0098908 (PMC4053352; doi:10.1371/journal.pone.0098908)

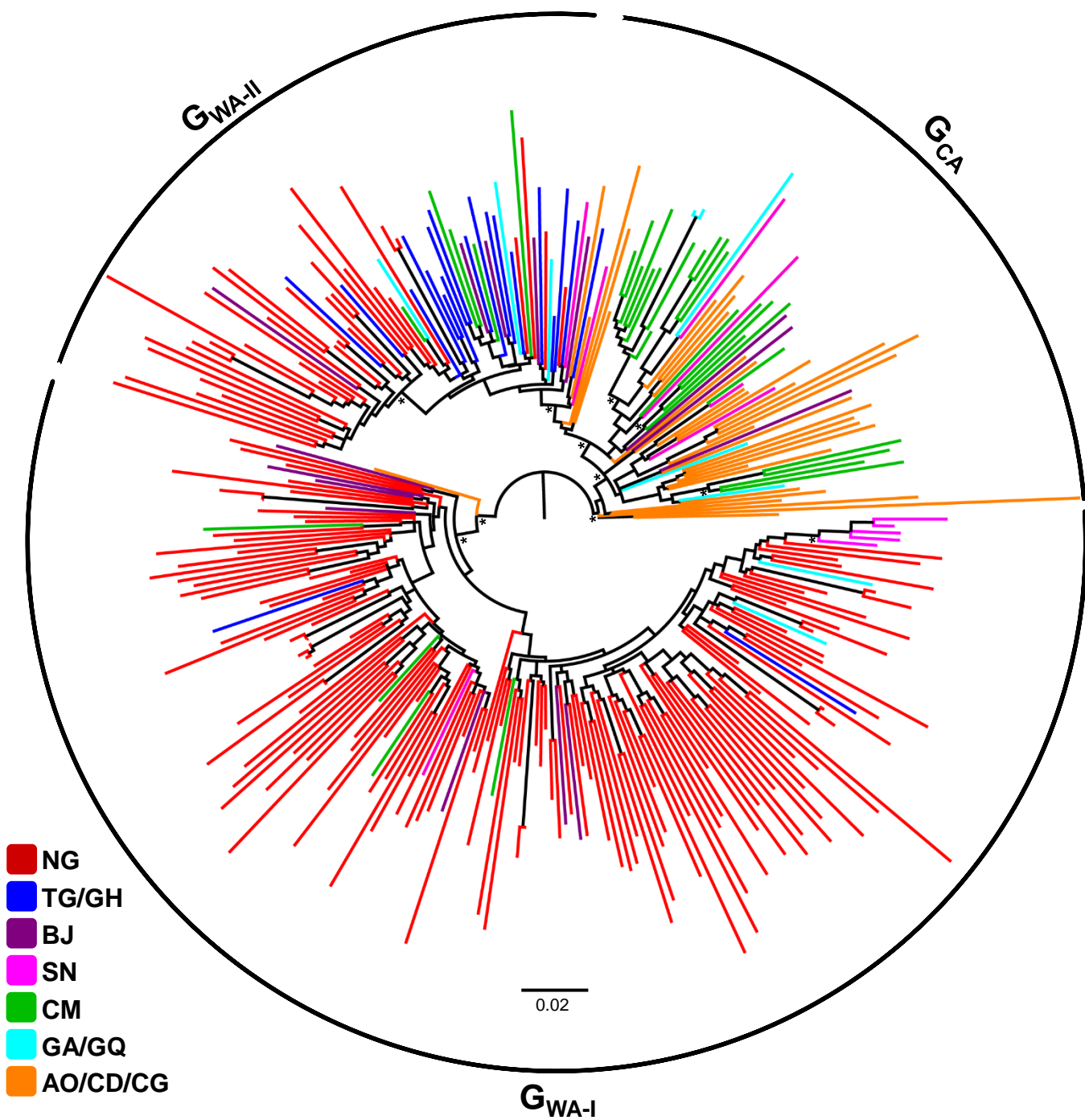

Figure S1

Supplement: Figure S1 — ML tree of the of the HIV-1 subtype G pol PR/RT sequences (∼1,000 nt) circulating in West and Central Africa. Branches are colored according to the geographic origin of each sequence as indicated at the legend (bottom left). Arcs indicate the positions of major subtype G clades characteristic of western (GWA-I and GWA-II) and central (GCA) African regions. Asterisks point to key nodes with high support (aLRT>0.85). The tree was rooted on midpoint. The branch lengths are drawn to scale with the bar at the bottom indicating nucleotide substitutions per site. (PDF) [file pone.0098908.s001.pdf]

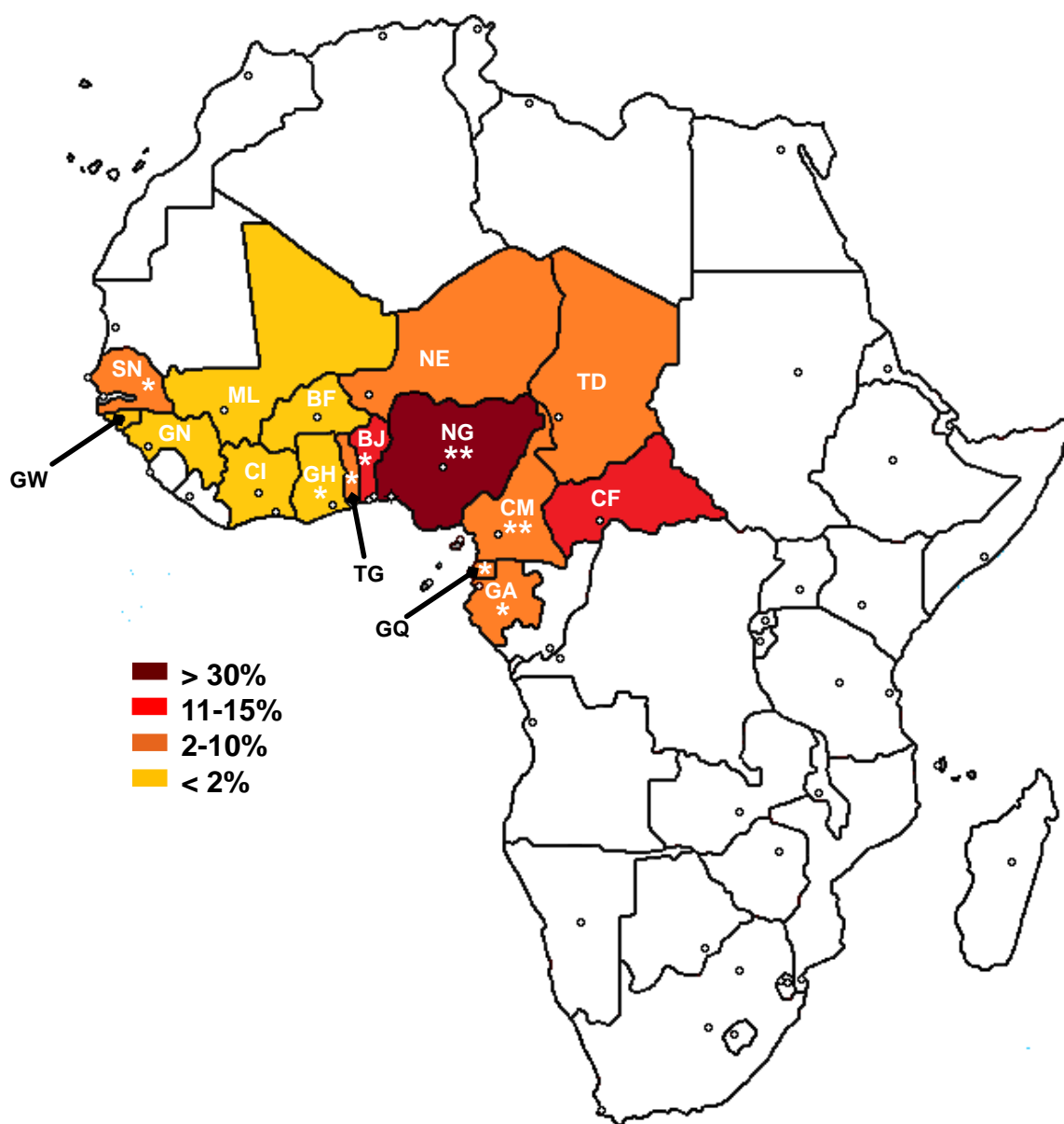

Figure S2

Supplement: Figure S2 — African map showing the prevalence of subtype G among HIV-1-infected individuals from West and West Central Africa, and the corresponding representativeness of each African country in our subtype G dataset. Countries were colored according to the relative prevalence of subtype G (estimated from references 5–30 and 53–58) as shown in the legend. Asterisks indicate countries represented by very high (***n>100), relatively high (**n>30), and small (*n≤30) number of sequences. Countries with no asterisks were not represented in our dataset. (PDF) [file pone.0098908.s002.pdf]
