# Supplementary material for: Spatiotemporal Dynamics of the HIV-1 Subtype G Epidemic in West and Central Africa
Source: PLoS One. 2014 Jun 11;9(6):e98908. doi: 10.1371/journal.pone.0098908 (PMC4053352; doi:10.1371/journal.pone.0098908)
Supplement: Table S1 — Number of viral migration between locations estimated using Markov jumps counts. (PDF) [file pone.0098908.s003.pdf]

Table S1. Number of viral migration between locations estimated using Markov jumps counts.

From	To						
	AO/CD/CG	CM	GA/GQ	BJ	GH/TG	NG	SN
AO/CD/CG	-	6.1	2.3	2.2	1.5	1.2	4.2
CM	1.2	-	2.6	0.5	0.6	0.2	1.2
GA/GQ	0.2	0.8	-	0.5	0.3	0.1	0.5
BJ	0.2	0.4	0.7	-	0.9	0.5	0.5
NG	0.4	4.4	2.3	8.1	5.5	-	1.9
SN	0.4	0.8	0.6	0.4	0.2	0.1	-
TG/GH	0.3	3.9	2.3	5.7	-	4.1	1.0

AO/CD/CG: Angola/Democratic Republic of Congo/Republic of Congo. CM: Cameroon. GA/GQ: Gabon/Equatorial Guinea. BJ: Benin. NG: Nigeria. SN: Senegal. TG/GH: Togo/Ghana.
