# Supplementary material for: Spatiotemporal Dynamics of the HIV-1 Subtype G Epidemic in West and Central Africa
Source: PLoS One. 2014 Jun 11;9(6):e98908. doi: 10.1371/journal.pone.0098908 (PMC4053352; doi:10.1371/journal.pone.0098908)
Supplement: Table S2 — Evolutionary rate and time-scale of HIV-1 subtype G and major regional clades circulating in Africa. (PDF) [file pone.0098908.s004.pdf]

Table S2. Evolutionary rate and time-scale of HIV-1 subtype G and major regional clades circulating in Africa.

**Clade**	***N***	***μ* (subst./site/year)**	**Coefficient of variation**	**T_{MRCA}**
Subtype G	305	2.3×10^{-3} ($1.8 \times 10^{-3} – 2.8 \times 10^{-3}$)	0.28 (0.24-0.32)	1968 (1956-1976)
G_{WA-I}	167	2.6×10^{-3} ($2.1 \times 10^{-3} – 2.9 \times 10^{-3}$)[a]	0.28 (0.22-0.33)	1978 (1970-1983)
G_{WA-II}	70	2.6×10^{-3} ($1.9 \times 10^{-3} – 2.3 \times 10^{-3}$)[a]	0.25 (0.17-0.34)	1984 (1980-1987)
G_{CA}	60	2.6×10^{-3} ($1.8 \times 10^{-3} – 2.3 \times 10^{-3}$)[a]	0.30 (0.20-0.39)	1975 (1970-1981)

[a] Estimations obtained using an informative prior for clock rate equal to the posterior distribution estimated from the global dataset ($1.8 \times 10^{-3} – 2.8 \times 10^{-3}$ subst./site/year).
