# Supplementary material for: Spatiotemporal Dynamics of the HIV-1 Subtype G Epidemic in West and Central Africa
Source: PLoS One. 2014 Jun 11;9(6):e98908. doi: 10.1371/journal.pone.0098908 (PMC4053352; doi:10.1371/journal.pone.0098908)
Supplement: Table S3 — Best fit demographic model for HIV-1 subtype G African clades. (PDF) [file pone.0098908.s005.pdf]

**Table S3.** Best fit demographic model for HIV-1 subtype G African clades.

| Clade | Model | PS Log ML | Models compared | Log BF | SS Log ML | Models compared | Log BF |
|---|---|---|---|---|---|---|---|
| $G_{WA-I}$ | **Log** | **-21511.6** | - | - | **-21513.1** | - | - |
| | Expo | -21597.4 | Log/Expo | 85.8 | -21599.1 | Log/Expo | 86.0 |
| | Expa | -21604.2 | Log/Expa | 92.6 | -21605.8 | Log/Expa | 92.7 |
| $G_{WA-II}$ | **Log** | **-10000.2** | - | - | **-10000.6** | - | - |
| | Expo | -10039.7 | Log/Expo | 39.5 | -10039.7 | Log/Expo | 39.1 |
| | Expa | -10046.3 | Log/Expa | 46.1 | -10046.5 | Log/Expa | 45.9 |
| $G_{CA}$ | **Log** | **-9434.9** | - | - | **-9435.2** | - | - |
| | Expo | -9450.4 | Log/Expo | 15.5 | -9450.7 | Log/Expo | 15.5 |
| | Expa | -9456.8 | Log/Expa | 21.9 | -9457.0 | Log/Expa | 21.8 |

Log marginal likelihood (ML) estimates for the logistic (Log), exponential (Expo) and expansion (Expa) growth demographic models obtained using the path sampling (PS) and stepping-stone sampling (SS) methods. The Log Bayes factor (BF) is the difference of the Log ML between of alternative (H1) and null (H0) models (H1/H0). Log BFs > 3 indicates that model H1 is more strongly supported by the data than model H0. The best fit model for each data set is indicated in bold.
